# An action‐oriented framework for systems‐based solutions aimed at childhood obesity prevention in US Latin*x* and Latin American populations

**DOI:** 10.1111/obr.13241

**Published:** 2021-04-07

**Authors:** Leandro M. T. Garcia, Ruth F. Hunter, Kayla de la Haye, Christina D. Economos, Abby C. King

**Affiliations:** ^1^ Centre for Public Health Queen's University Belfast Belfast UK; ^2^ Keck School of Medicine University of Southern California Los Angeles California USA; ^3^ Friedman School of Nutrition Science and Policy Tufts University Medford Massachusetts USA; ^4^ Department of Epidemiology and Population Health Stanford University School of Medicine Stanford California USA; ^5^ Stanford Prevention Research Center, Department of Medicine Stanford University School of Medicine Stanford California USA

**Keywords:** complex interventions, obesity prevention, systems science, young people

## Abstract

Childhood obesity in US Latin*x* and Latin American populations is a persistent, complex public health issue and, as such, requires solutions grounded on systems science theory and methods. In this paper, we introduce an action‐oriented framework to design, implement, evaluate, and sustain whole‐of‐community systems changes for childhood obesity prevention in US Latin*x* and Latin American populations. Our framework covers six action steps: (1) foster multisectoral team; (2) map the system, its context, and drivers; (3) envision system‐wide changes; (4) effect system‐wide changes; (5) monitor, learn, and adapt; and (6) scale and sustain. We also propose 10 principles that put human and environmental rights and systems thinking at the center of these systems‐based solutions. For each action step, we provide a list of concrete activities, methods, approaches, and examples that can be used to guide and inform the work needed to achieve the expected outputs. Finally, we discuss how a wider adoption of systems science for childhood obesity prevention among US Latin*x* and Latin American populations can be encouraged and sustained.

## 1 INTRODUCTION

It is well recognized that rising levels of childhood obesity in US Latin*x* and Latin American populations is a persistent, complex problem with important implications for health and beyond. It has the characteristics of a complex problem in that it is driven by influential factors and social actors (e.g., people and organizations) operating and dynamically interacting across multiple levels over time.^1^, ^2^, ^3^, ^4^ It therefore follows that childhood obesity prevention efforts will need to operate within this complex adaptive system and, as such, could attain better results with the application of systems science theory and methods.^5^

Systems science is an interdisciplinary field engaged in the study of the properties of systems—integrated wholes made up by interdependent elements. Complex adaptive systems are special cases of systems: they are formed by many elements or components that are irreducibly entwined, interacting over time with no or low central coordination or control, and they create patterns and ways of functioning that are not displayed by the individual components and that adapt in response to changes in the context in which the system exists.^6^ The levels of childhood obesity in US Latin*x* and Latin American populations can be conceptualized as the result of a complex adaptive system encompassing the food, school, and transport systems, sociocultural and environmental influences, and numerous other factors that interact to shape energy intake and expenditure.^4^

For instance, Pérez‐Escamilla et al.^1^ searched for elements of complex adaptive systems in successful initiatives related to childhood obesity prevention in Latin America, namely, sugar sweetened beverages and junk food taxes in Mexico, front of package food labeling in Chile and Ecuador, reduction of trans fatty acids in food supply in Argentina, and the open streets program known as *Ciclovías Recreativas* in Colombia. The authors found that several key factors associated with the successes observed across these case studies were those that addressed properties of complex systems. These factors included consideration of feedback loops that facilitate or obstruct implementation or continuation of a policy; exploitation of characteristics of social networks that influence the diffusion of knowledge and behaviors; facilitation of radical changes in the systems as they reach a tipping point; and consideration of historical context and the collective patterns and behaviors that emerge from the interaction between the systems' elements.

Implementing changes to how a complex adaptive system is structured and operates can be understood as effecting a disruption—a threat, to some extent—to the current form and function of the system. Complex systems tend to settle in stable states and resist or overcome disruptions that push them toward a new regime.^7^ That means that efforts to reconfigure the system tend to trigger the system's responses to the intervention itself, oftentimes resulting in failure to address the problem, an issue known as resistance to change (or intervention or policy resistance).^8^

Calls to expand the application of systems thinking in efforts to prevent childhood obesity in US Latin*x* and Latin American populations have been increasing rapidly. However, much of the current literature is based on theory rather than practical applications.^2^, ^9^, ^10^ There is limited guidance for interdisciplinary and multisectoral teams (e.g., public health professionals, professionals from other sectors, evaluators, academics, community leaders, decision‐makers, and stakeholders) willing to develop system‐based solutions to address childhood obesity in their communities.

In this paper, we introduce an action‐oriented framework that addresses the need for a more concrete roadmap to incorporate and use systems science theory and methods to design, implement, evaluate, and sustain whole‐of‐community systems changes for childhood obesity prevention in US Latin*x* and Latin American populations.

In Table 1 you will find a glossary of systems science concepts present in the next sections. These terms are indicated in italics throughout the text.

**TABLE 1:** Glossary of systems science concepts

*Adaptive capacity:* capacity to adjust its own characteristics or behaviors to respond to existing and future conditions and problems.^11^ *Agent‐based model:* quantitative modeling technique that simulates a composition of autonomous entities (e.g., persons or organizations), called agents, that “make decisions” according to a set of behavioral rules that guide their interaction with the surrounding environment and other agents over time, shaping the system's global patterns.^12^ *Complex adaptive system:* system formed by many components that are irreducibly entwined, interacting over time with no or minimal central coordination or control, creating collective patterns and ways of functioning that are not displayed by the individual components and that adapt in response to changes in the context the system exists.^6^ *Feedback loop:* cause‐and‐effect chain that connects two or more factors in a circuit (or loop). Feedback loops can be positive (factors reinforce each other over time) or negative (factors balance each other over time).^13^ *Group model building:* participatory method for involving people in a modeling process.^14^ *Just‐in‐time adaptive intervention:* intervention design that adapts its properties (e.g., the type, timing, intensity) as conditions and contexts change, delivering support at the moment and in the context that it is most needed or is most likely to be receptive.^15^ *Network analysis:* set of methods and techniques to describe and analyze networks, i.e., structures of relations or connections among entities (e.g., people, organizations, projects).^16^ *Self‐organization:* process through which a system's global structure arise solely from local interactions among the elements of the system, with no or minimal central coordination or control.^6^ *System‐based solution:* processes and actions to deal with a problem that are underpinned by systems thinking and systems science principles and methods. *System dynamics model:* quantitative modeling technique that uses coupled differential or integral equations to describe and analyze the global behavior of complex systems over time. These equations represent the factors and quantities involved in the systems and how they affect each other over time.^13^ *Systems science:* interdisciplinary field engaged in the study of the properties of systems.^6^ *Systems thinking:* way of thinking, conceptualize, and make sense of the world characterized by the application of core systems concepts (e.g., inter‐relationships, feedback loops, adaptation, self‐organization).^17^

## 2 AN ACTION‐ORIENTED FRAMEWORK FOR SYSTEMS CHANGES FOR CHILDHOOD OBESITY PREVENTION

**FIGURE 1:**
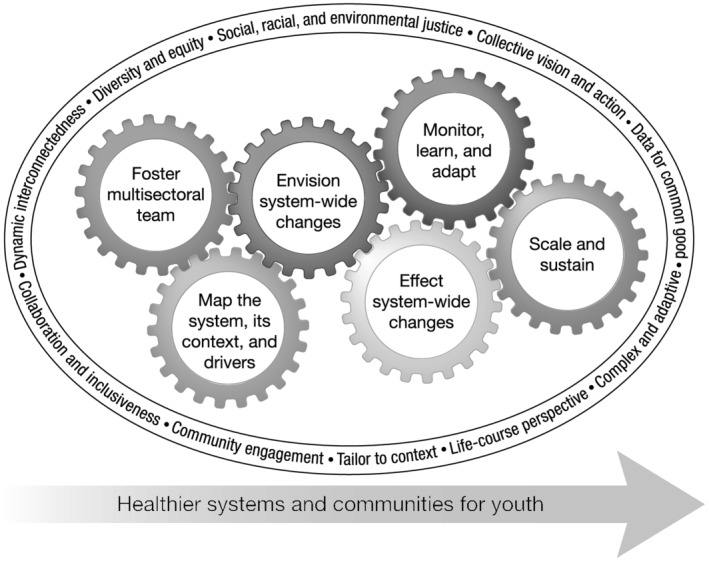
Action‐oriented framework for systems‐based solutions for childhood obesity prevention in US Latin*x* and Latin American populations

**FIGURE 2:**
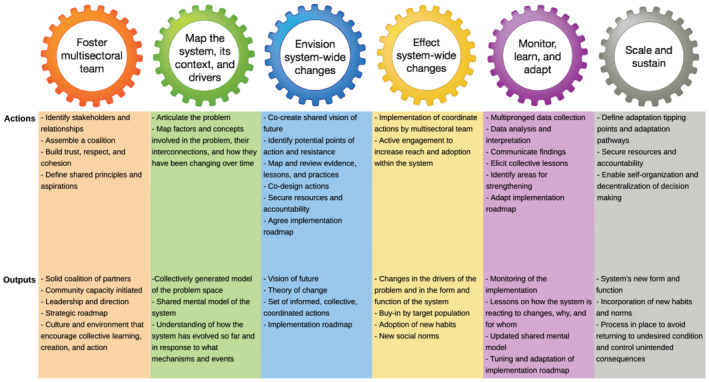
Actions and expected outputs of each action step in the framework

**FIGURE 3:**
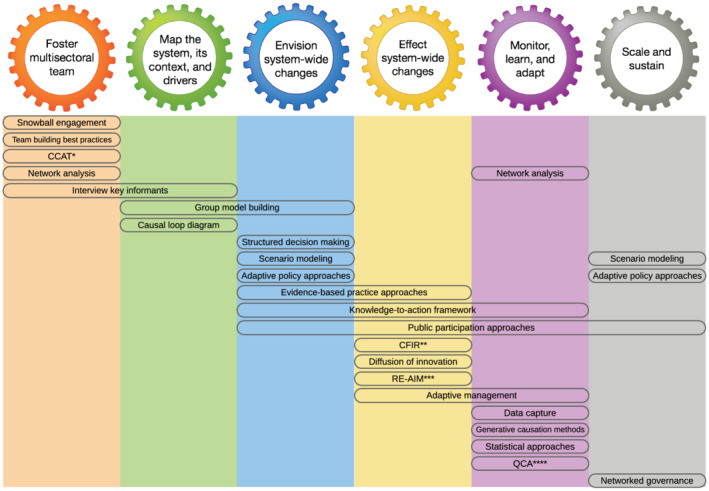
Examples of methods and approaches for each action step. ^*^Community coalition action theory. ^**^Consolidated framework for implementation research. ^***^Reach, effectiveness, adoption, implementation, and maintenance (RE‐AIM) framework. ^****^Qualitative comparative analysis

Our action‐oriented framework covers the activities that are critical to achieve sustained changes in systems that fuel and perpetuate childhood obesity in US Latin*x* and Latin American populations. In our framework (Figures 1, 2, 3 and Table S1), each activity is represented by a different action step (represented in the figures as “cogs”) which together comprise the overall framework, as follows: creating and nurturing a multisectoral team (orange cog); understanding the system and the underlying causes of the problem (green cog); envisioning system‐wide changes (blue cog); transforming vision into action through coordinated actions to change how the system operates (yellow cog); monitoring how the actions are implemented, understanding how the system as a whole and its various elements respond and adapting actions accordingly (purple cog); and identifying ways to scale and sustain system‐wide changes (gray cog).

As illustrated in Figure 1, a *system‐based solution* to reduce childhood obesity in US Latin*x* and Latin American populations is composed of the above activities that interact with each other over time in direct and indirect ways and play a fundamental role in the success of the solution. Obstructions to the execution of any one activity, such as delaying action or depriving required resources, can cause negative ripple effects for the other activities and, consequently, impact the extent to which system‐wide changes can be achieved and sustained.

Helping interdisciplinary teams develop *system‐based solutions* to reduce childhood obesity in their communities is at the heart of our framework. To help with this goal, for each of the activities, we provide a non‐exhaustive list of concrete actions (Figure 2 and Table S1) and methods and approaches (Figure 3 and Table S1) that can be used to guide and inform the work needed to achieve the expected outputs. The subsequent sections expand on each of these points and provide some examples.

We further propose that *system‐based solutions* to reduce childhood obesity in US Latin*x* and Latin American populations should be underpinned by the following 10 principles that put human and environmental rights and *systems thinking* at the center of these solutions (Figure 1), as follows:

1. Dynamic interconnectedness: factors and mechanisms at different levels (individual, interpersonal, organizational social, environmental, economic, policy, and planetary) are interconnected, influencing each other directly or indirectly, leading to a cycle of influence over time (i.e., feedback structures).
2. Complex and adaptive: the form and function of the system as a unit emerge and adapt from the network of dynamic interactions between people, organizations, and environments that compose the system. For instance, how stakeholder groups are formed and function play a critical role in the success of community‐wide childhood obesity prevention initiatives.^18^, ^19^
3. Tailor to context: the form and function of *complex adaptive systems* respond and adapt to the larger context in which they exist. For instance, changes in lobby and marketing strategies of transnational food industries can affect the availability of unhealthy foods in US Latin*x* and Latin American communities. Furthermore, it is important that we have data systems that can monitor changes in the larger context and facilitate timely adaptation measures to continually adjust and optimize the system's response and outcomes.
4. Life‐course perspective: childhood obesity prevention efforts should be designed and implemented based on the scientific evidence of risk and protective factors across different developmental stages and needs across the life course. Evidence shows that the time between conception and 5 years of age is a critical window for obesity prevention throughout life, with caregiver‐child feeding interactional patterns having crucial contribution on lifelong healthy weight‐related behaviors.^20^ Also, sustainable solutions should be adaptable to changes over the life‐course in society at large,^4^ such as the availability of ultra‐processed food.
5. Diversity and equity: intentional inclusion of the perspectives of multiple sectors, population groups, and stakeholders is critical to correct for imbalances in power, privilege, and influence arising, for instance, from structural and historical social disparities and discrimination. Prejudice and stigma against US Latin*x* and Latin American groups can result in under‐representation of their perspectives in the development of the interventions. Moreover, some population groups within the communities are even more affected by these imbalances, such as girls, women, and members of the LGBTQ+ community.
6. Social, racial/ethnic, and environmental justice: fair treatment, equal opportunities, meaningful involvement, and the same degree of protection from environmental and health hazards should be provided to all members of the population being served by the initiative. There should be a focus on activities that serve to address injustices and inequities instead of activities that maintain the *status quo* or exacerbate these issues. US Latin*x* populations are more likely to live in neighborhoods with less opportunities for physical activity and healthy diet and more environmental risks because of systemic structural racism,^21^ and within these communities, some might be more severely impacted, such as young children and people with disabilities.
7. Collective vision and action: all relevant stakeholders should be engaged in the development of a shared understanding of the problem, a shared vision of the future, and a plan to take collective action to move the system toward a more desirable and optimal state for all. Otherwise, individual stakeholders will keep working as individuals, oriented only by their own goals and perspectives without the wider coordination necessary to overcome the *status quo*.
8. Community engagement: the population being served by the initiative must be involved in the planning, execution, translation, and sustainability of activities in a meaningful way. Community engagement raises and empowers the members of the US Latin*x* and Latin American communities being served as partners in the project team, leading to positive impacts on design, implementation, and receptiveness of the interventions.^22^
9. Collaboration and inclusiveness: all relevant stakeholders should be respected, given the opportunity and space to be heard and participate in a safe and meaningful way throughout the activities.
10. Data for common good: data that can be used for the benefit of the system should be made accessible and usable by all relevant stakeholders, facilitating the collective planning, execution, and monitoring of actions.

In the next sections, we provide an overview of each action step of this framework.

### 2.1 Foster a multisectoral team

A critical starting point for initiating system level change to prevent childhood obesity is to identify and meaningfully engage with the key people, organizations, and social actors who will play a role in understanding, changing, and sustaining local systems. For childhood obesity, this may involve engaging with a core team, and broader coalition, that includes leaders from the Latin*x* community, as well as parents, teachers, coaches, child health care providers, business, government, non‐profit, and scientific leaders who have an interest in, or influence on, child wellbeing.^23^ Without the careful identification of this breadth of stakeholders, the team is likely to have an incomplete perspective of the system and factors that impact child obesity, because people with differing perspectives and roles in system were not involved in articulating the problem and the underlying social determinants.^24^ Engaging a diverse team is also needed to create a vision for the future; identify the best actions that are aligned with local priorities, capacity, and resources; and foster long‐term buy‐in and support needed for sustained community adoption.

Building this multisectoral team should begin by “starting where the people are”^25^ and identifying key actors and trusted relationships that are committed to and/or engaged in the issue of childhood obesity.^23^ Additionally, it is helpful to engage with people and organizations that play a gatekeeping or “bridging” role between health care or scientific team members and the community,^24^ such as community organizers or liaisons who understand the Latin*x* community being served and are viewed as credible and trusted.^26^ In addition, employing systematic methods for including the voices of community residents themselves, critical to enrich the information being gathered and acted upon, is been increasingly recognized.^27^, ^28^ The scientific members of the team should also be multidisciplinary, ideally integrating expertise in health and medicine, child development, social and cultural sciences, health policy, community psychology, data/analytic sciences, and systems science. Finally, a snowball engagement approach,^29^ where initial team members and community leaders invite other stakeholders they know to get involved, can help to ensure there has been adequate reach.

Critical for implementing policy changes is the meaningful involvement of government actors, as well as the consideration of the characteristics, barriers, and facilitators of the political decision‐making process. For instance, investigating the dynamics of obesity prevention policy decision‐making of the Healthy Together Victoria initiative (a state government‐led, multi‐level, multi‐setting complex systems approach to obesity prevention in Australia), Clarke et al.^30^ identified that alignment of policy proposals to other government objectives and development of viable policy solutions that met the requirements and beliefs of decision‐makers were important facilitators for policy adoption. In contrast, the organizational culture of risk aversion and time required for the policy process can create barriers and delays in the policy decision‐making process.

A useful step in fostering a multisectoral team is to map the network of relationships, such as existing collaborations, that exist among the members. Social *network analysis* is a useful tool for this^16^ because it can provide insights into clusters of stakeholders that are already well connected or disconnected and identify important opinion leaders who are known by many in the team and who could provide leadership.^31^ It also can be used to help make the coalitions more efficient, effective, and sustainable by employing network intervention strategies.^32^

Intentional activities are needed to solidify the team as a collective who will work together on shared issues and goals.^24^ Community Coalition Action Theory^33^ provides useful guidance on this process, emphasizing that team/coalition members should perceive a need to belong to the group and that the benefits of being involved (e.g., access to information and increased capacity to address a valued issue) outweigh the costs (e.g., time and resources). An example is the Shape Up Under 5, a whole‐of‐community childhood obesity prevention intervention conducted in Sommerville, Massachusetts, United States, that convened 16 stakeholders from six different sectors (early education and care, parks and recreation, the local health department, healthcare, food assistance programs, and the Somerville Public Schools) as equal partners working together in the design, implementation, and evaluation of the intervention.^19^ Some of the activities used to build and sustain the coalition were monthly facilitated meetings, evidence, and resource sharing for mutual learning and dialog and *group model building* exercises.

Team and coalition building can be assisted by having the stakeholders engage in frequent communication, and setting up clear leadership roles as well as group processes that involve shared decision‐making.^34^ Members' knowledge and skills should not be viewed as a hierarchy but, instead, coming from multiple sources where different stakeholders bring valuable strengths and perspectives. In US Latin*x* and Latin American communities, it is also important to consider existing cultural and historical perspectives of the community stakeholders and to recognize and actively work to correct power, privilege, and influence imbalances that may be based on structural inequalities and discrimination.^35^ Scientific team members may have unique barriers to engagement that need to be addressed, such as a lack of knowledge about the benefits of multisectoral research and a lack of institutional support for, or recognition of, community‐based research and team‐building activities.^24^

A contentious point in the building of multisectoral teams for childhood obesity prevention initiatives is the participation of opposing forces, the food and beverage industry in particular, which has been involved in practices that increase children's risk for obesity and market unhealthy foods to communities with disadvantage, including Latin*x*, in favor of maximizing profit.^36^ In our view, all actors who play a role in the form and function of the system should be identified and invited to productively contribute to the multisectoral team. However, those who fail to align with the team's vision, goals, culture, and environment may need to be disengaged from the team before the relationship becomes more of a liability than an asset. The ongoing presence of combative voices brings implications in terms of influence, trust, and management of conflicts of interest within the team and in the eyes of the public; thus, those leading the team should have strategies in place to diminish the probability of such deleterious impacts occurring through appropriate structuring of expectations and goals up front, in addition to dealing effectively with such issues should they arise.

Altogether, these activities can be used to foster a representative and multisectoral team of stakeholders who are engaged in creating systems change to address childhood obesity. These activities are intended to help foster a team and broader coalition that has a culture and environment that will encourage bi‐directional instead of top‐down relationships, and a space for collective knowledge creation, learning, and actions that will be essential qualities to support the team in their subsequent phases of action.

### 2.2 Map the system, its context, and drivers

Central to a systems‐based solution to reduce childhood obesity is to understand how factors at multiple levels (individual, interpersonal, organizational, social, environmental, economic, and policy‐based) and actors across sectors operate and dynamically interact, shaping and sustaining the system and the targeted problem. A clear understanding of the system and the larger context in which it exists is required to design and effect sustained changes in the system.

It is not unusual to react to complexity by either ignoring it or reducing it to simple, unidirectional cause‐effect patterns, ignoring *feedback loops*, disregarding the significance of delays between causes and effects, and making mistakes about effects when there are two or more causes interacting.^2^, ^14^, ^17^, ^37^ Moreover, actors contained in a system usually operate only at certain parts of the system, lacking full understanding of the various factors, mechanisms, and interactions operating in the entire system. That means that their individual mental models of how the system operates are often flawed.^13^, ^14^, ^37^ Therefore, collectively generated problem articulation and mapping of the system are key to inform the design, implementation, assessment, and sustainability of systems change efforts to reduce childhood obesity in US Latin*x* and Latin American populations.

Frameworks and methods rooted in *systems thinking* principles exist to help multisectoral teams externalize their mental models and shift from an individual to a communal perspective of the system and the targeted problem. This often involves employing *group model building* approaches, such as soft systems methodology (a problem structuring method),^38^, ^39^ community‐based *system dynamics*,^14^ and the Foster‐Fishman et al.^37^ framework for characterizing systems change, among others. In general, the first step for the multisectoral team is to define the problem, that is, the fundamental system‐wide issue that will be targeted. An example of a system‐wide issue is the level of obesity among school‐aged children in a US Latin*x* community. Drawing the system's “behavior” over time (e.g., the observed levels of childhood obesity over the last 10 years and expectations or predictions for the next 10 years) alongside the best and worst scenarios helps to frame the problem and envision the magnitude and timeframe of the desired change. A variety of other activities to engage the group in understanding the problem and the variables within the system are often employed, including connection circles, impact/feasibility grids, and initial (and simple) diagrams connecting variables.

Next is the mapping of factors, actors, processes, and contextual elements driving the levels of childhood obesity.^2^, ^14^, ^37^ The goal is to draw a diagram of the system that explains the levels of childhood obesity as an endogenous consequence of the feedback structure between factors.^13^, ^14^, ^37^ Causal loop diagrams are one of the most widely used tools for this end.^13^ They consist of variables connected by arrows that denote the causal influence among the variables, allowing the identification of *feedback loops*. These diagrams are useful for eliciting and capturing mental models of individuals or teams and facilitating knowledge sharing.^13^ For instance, the Shape‐Up Somerville project used causal loop diagrams to map the factors and feedback structures that could impact the success of its whole‐of‐community childhood obesity prevention intervention, identifying factors in eight subsystems or domains (individual, family, school, built environment, food environment, community, Shape Up Somerville Task Force, and media), illustrating the interplay with and between subsystems and how they come together as a whole system.^9^

It is important to acknowledge that knowledge about the system's form and function and the potential drivers of the levels of childhood obesity is distributed among the actors contained in the system. This underscores the critical need to engage a broad range of stakeholders relevant to the problem and mobilize their viewpoints and knowledge to achieve a better understanding of the system as a whole. Interviews with key informants and *group model building* activities are two ways of engaging with stakeholders, the latter enabling their active participation in the process of developing the systems map, leading not only to a more relevant model, but to a communal understanding of the system's form and function and shared vision and insights.^2^, ^9^, ^14^, ^37^, ^39^, ^40^

### 2.3 Envision system‐wide changes

Once the multisectoral team has a shared articulation of the problem and understanding of the system, its context, and putative drivers, the next step is to co‐create a shared vision of the future. This action step is integral to the success of the entire initiative, given that without the true co‐creation of a shared way forward, any suggested initiatives and actions taken are more likely to fail, suffer delays, and be less effective and unsustainable. This action step will benefit from drawing on the approaches and lessons in implementation science exemplified in this series.^41^

Steps to co‐creating a shared vision include (a) identifying potential points of action, leverage points, and points of resistance to change in addressing childhood obesity in US Latin*x* and Latin American populations; (b) mapping current and planned local actions; (c) reviewing evidence‐informed practices and practice‐based evidence specific to US Latin*x* and Latin American populations; (d) co‐designing actions that are acceptable, feasible, effective, adaptable, and sustainable to US Latin*x* and Latin American communities, taking account of important social and cultural considerations; (e) securing resources and accountability for implementation^41^; and (f) defining steps, actors, roles, resources, timeline, and checkpoints for implementation.^41^

Key outputs from this activity include the development of and agreement on a shared vision and roadmap to action that, importantly, is equitable and inclusive. These outputs are the usual subsequent steps of the process initiated by mapping the system, particularly when *group model building* methods are used.^2^, ^14^, ^39^ The articulation of an implementation roadmap can be facilitated by structured decision‐making approaches,^42^ widely used in environmental management but that can be easily adapted to aid and inform decisions, plans, and actions in public health. When developing the implementation roadmap, it is important to include consideration of both the intended/unintended and anticipated/unanticipated consequences of actions^42^, ^43^ and acknowledge the different priorities and interests of participating stakeholders.^10^, ^14^, ^39^, ^42^ Systems approaches are well suited to unveil and address unintended and unanticipated impacts of childhood obesity prevention initiatives that can arise from feedback structures and flawed mental models of how the system operates. For instance, the implementation of a new bicycle sharing system for children to encourage active travel between home and school without the provision of new traffic safety measures can result in increased rate of road collisions and injuries involving children, increased risk perceived by parents and children, and reduction in children's cycling behavior both for transport and recreation, decreasing the demand and support for the bicycle sharing system.

Approaches such as Evidence‐Based Practice^44^ and Knowledge‐to‐Action Framework^45^ can inform the identification, evaluation, and adaptation of local and external evidence to implement the best set of actions for local context, including promising macro‐environment and systems change approaches. These approaches can guide the accumulation and analysis of the best evidence and knowledge available to inform the development and implementation of childhood obesity prevention initiatives. Scenario modeling methods, such as *system dynamics* and *agent‐based modeling*, are particularly helpful to work through and compare different types of strategies, actions, outputs, and outcomes in complex situations and contexts.^9^, ^13^, ^14^, ^46^

A final output from this activity is the co‐development of a set of informed, collective, coordinated actions that are robust, yet adaptable to the uncertainties, and future contextual changes and disruptions. An articulated theory of change^47^ that describes the pathway or sequence of steps in getting from the initiatives' activities to the expected impacts and the causal assumption behind the links in the pathway can help provide a framework to support the implementation roadmap, designing a set of interventions that work at multiple levels and over multiple timeframes. The need to be flexible and malleable can be facilitated by adaptive policy approaches (see more about these approaches in Section 2.6).^48^, ^49^, ^50^ Public participation approaches (e.g., citizen panels) can be used to ensure that articulated actions are acceptable, feasible, effective, adaptable, and sustainable from the viewpoint of the population being served by the initiative.

### 2.4 Effect system‐wide changes

Interventions, such as those required to tackle childhood obesity in US Latin*x* and Latin American populations, can be envisioned as disruptions in *complex adaptive systems*. At this stage, the multisectoral team implements the system‐wide changes that they have envisioned in the previous steps, and specific actions they have agreed upon, to achieve this change. These activities should be executed by the team and their collaborators, bringing in new expertise as needed, and coordinated through regular team meetings and ongoing stakeholder feedback. Team members' active engagement with broader community members and stakeholders throughout this phase will help to increase buy‐in by the community being served, as well as the reach and sustainability of these actions.

Actions should be supported by evidence‐based principles of dissemination and implementation science to accelerate and maximize their impact on the system and population being served by the intervention. Dissemination and implementation science identifies several strategies and factors that have been shown to increase the speed at which public health actions and interventions are adopted, and the extent to which they are effective and sustained.^51^

Rogers's Diffusion of Innovations^52^ is one widely used theory that provides useful insights into how to increase the adoption and spread of new ideas, practices, and behaviors in the population being served. In public health interventions broadly, and childhood obesity interventions specifically, the adoption of new practices and health behaviors (e.g., healthier eating and physical activity habits) is often slow and not sustained. Diffusion of Innovations theory points to several characteristics of the innovation, of the “adopters”, and of the system in which adoption is occurring, that can be addressed to help accelerate system‐wide adoption. For example, system‐wide changes to address childhood obesity in US Latin*x* and Latin American populations will more likely occur and be sustained if the programmatic actions (e.g., changes to school lunches) and individual behaviors (e.g., walking to school) to be adopted (i) are not too complex; (ii) are compatible with the communities' values and goals; (iii) can, when taken up by “early adopters”, be observed by others (who may be contemplating adoption); and (iv) are something that people and organizations feel that they can “try out” (e.g., make an initial change for a short period of time). Focusing on getting opinion leaders among the stakeholders and in the community being served to be the initial adopters of these actions and behaviors can also increase adoption and buy‐in by others.

The Reach, Effectiveness, Adoption, Implementation, and Maintenance (RE‐AIM) framework also provides guidance to improve the translation of interventions and actions into effective system‐wide change.^41^ This includes strategies to increase the number and breadth of people in the population being served who are exposed to the actions (Reach), optimizing the positive health changes that happen when exposed to the actions (Effectiveness), increasing uptake of the actions and interventions by key actors in the systems (Adoption), ensure faithful and effective deliver of the actions and interventions (Implementation), and ways to increase the likelihood that the actions and changes are sustained (Maintenance).

The results of the actions implemented by the team are intended to create meaningful change in multiple drivers of childhood obesity that were identified by the team. As a result, processes and features of the complex system that influences childhood obesity risk will begin to change, thereby changing the form and function of the system. And as the system changes, the resultant effects should be changes in children's health behaviors, leading to longer‐term behavioral patterns and habits and biological changes, sustained by a system that increasingly enables children's engagement in healthy habits that reduce risk for obesity. The collective adoption of healthier habits among children and target actions by broader members of the population being served can result in a shift of social norms and practices, so that these behaviors are increasingly perceived as normative and become embedded in community routine. This normative shift can have a reinforcing, positive feedback effect on collective behavior change and support the ongoing intervention actions.

### 2.5 Monitor, learn, and adapt

Integral to *systems science* are the tools used to continually monitor implementation and progress, learn what is and is not working and why, and adapt the interventions to ensure that the goals can be achieved. This part of the process emphasizes learning while doing and enables adjustments over time to achieve optimal conditions, outputs, and outcomes. Key features of monitoring intervention implementation include learning lessons on how the system is reacting to changes, why, for whom, and under what circumstances. It facilitates continual updating of the shared mental model, which then in turn supports fine tuning and adaptation of the implementation activities.

Monitoring can be achieved through the integration of several methodologies. Monitoring the system, ideally in real‐time or near real‐time, requires multi‐pronged data collection, collation, and synthesis from various sources (e.g., surveys, environmental and personal sensors, biomarkers, secondary “big data”, environmental features, policy analysis, and social media), employing novel data linkage methods. Multi‐method approaches are required to monitor actions efficiently and effectively, and the proximal and distal system changes happening across different timeframes, including unintended consequences.

A real‐time, integrated data environment would then aid *just‐in‐time adaptive interventions*,^15^ monitoring of, for example, eating and physical activity behaviors and changes in societal norms around these behaviors over time and data modeling. However, to be meaningful and facilitate adjustments in the system, the data and modeling need to be accompanied by analysis, interpretation, and communication that elicit collective lessons to inform further changes in the system. Ongoing monitoring and reflection mean that we can identify areas to improve practices that may not be working well, and which should be modified or stopped, and accelerate actions that are working well. Thus, the shared vision set out in previous activities can be adapted as required. Also, scalability and sustainability of the envisioned system‐wide changes depend on adequate monitoring processes (see Section 2.6).

A number of methodologies and approaches can be applied to monitor and adjust systems changes in childhood obesity research, but the specific set of methods and approaches can differ between interventions and over time for the same intervention as its evaluation needs changes. The selection of methods must consider what aspects of the system and intervention the multisectoral team considers important to track to evaluate progress and adapt actions. For instance, *network analysis* and diagnostics can be used to develop and monitor strategies to improve the efficiency, effectiveness, and sustainability of team collaboration networks involved in the planning and implementation of childhood obesity interventions.^31^, ^32^ Generative causation approaches (e.g., realist evaluation and contribution analysis) can help to articulate the underlying mechanisms or processes of change through which childhood obesity prevention interventions generate impact and the role of contextual factors to observed results.^53^ When various instances of the intervention are being assessed (e.g., across multiple Latin American countries), qualitative comparative analysis can be used to systematically identify the set and configuration of factors and processes that seems sufficient for the success of the interventions in the case studies.^53^, ^54^ Statistical approaches (e.g., interrupted time series analysis and difference in differences analysis) can help to handle quantitative data (e.g., levels of physical activity behavior and energy intake), synthesized expediently to facilitate data‐, evidence‐, and stakeholder‐informed adaptations to actions and interventions. Participatory approaches facilitate the active participation of stakeholders and the population being served by the initiative in the gathering and analysis of the data, providing real‐time, multi‐perspective interpretation on various aspects of the intervention.^55^

Approaches such as the Knowledge‐to‐Action Framework^45^ and adaptive management^56^ provide processes and tools that can be used to ensure that the outputs and lessons from the monitoring process are fed back to the multisectoral team to adjust actions and interventions as necessary. However, to be able to implement the required adjustments, effective communication channels are needed to ensure that all members of the multisectoral team, including children, parents, teachers, child health care providers, local business and industry, government, and researchers, have timely access to interpretable data, information, and knowledge to inform their decisions and actions. Finally, it is important to acknowledge the evolving and cumulative nature of evidence and knowledge generation required in *systems science* and, therefore, to be prepared to adjust the evaluation tools and processes as the system, and evaluative needs, changes over time.

### 2.6 Scale and sustain

Intervention or policy resistance—the system's counter‐response to an intervention, resulting in failure to address the target problem^8^—can be attributed mainly to the lack of understanding of the *feedback loops* triggered by our decisions and actions.^8^ A central tenet to overcoming intervention or policy resistance and facilitating and sustaining the reconfiguration of the system is to preserve the resilience of desirable parts of the system while trying to overcome the persistence of harmful parts.^7^ Part of this work needs to be the development and implementation of new policies that shift conditions that were holding a system in a state that resulted in negative outcomes, which can help to sustain change over the long term. But additional actions may need to be taken to facilitate the system's transition to, and the permanence of, the envisioned new form and function, ensure the incorporation of new habits and norms, and avoid unintended consequences and tendencies to return to the previous state.

One of the steps we suggest is to define adaptation tipping points and adaptation pathways, central elements of the Dynamic Adaptive Policy Pathways approach.^49^, ^57^ An adaptation tipping point specifies the conditions under which an action or a constellation of actions will no longer meet the objectives, and, therefore, new actions will be needed.^49^, ^57^, ^58^ Adaptation pathways are a sequence of possible actions after a tipping point is reached.^49^, ^57^, ^59^ These pathways can be developed and explored via scenario modeling (e.g., *system dynamics* and *agent‐based models*) and/or more qualitatively using public participation approaches. The process of defining adaptation tipping points and adaptation pathways prompts those implementing *system‐based solutions* to reduce childhood obesity in US Latin*x* and Latin American populations to consider and plan built‐in triggers and mechanisms for adjustments to steer the course of action in response to intervention or policy resistance and sustain the system reconfiguration process.

Moreover, adaptive policy approaches in general, such as Dynamic Adaptive Policy Pathways, are designed to acknowledge, accommodate, and prepare for unforeseen changes in context, ensuring that goals can be achieved and sustained despite unanticipated issues.^48^, ^50^, ^57^, ^60^ These approaches outline principles, mechanisms, and tools that can help to scale and sustain *system‐based solutions* to reduce childhood obesity that are robust across a range of plausible futures and capable to cope with deep uncertainties generated by unpredictable changes in contextual factors over time, such as societal perspectives and preferences and stakeholders' interests.^48^, ^50^, ^59^, ^60^

As system‐wide changes are achieved, the multisectoral team should enable *self‐organization* and decentralization of the decision‐making and implementation process among those delivering the interventions locally, boosting the scalability and sustainability of the system's reconfiguration. This process has at least three major benefits^11^: (a) it can help make the most of stakeholders' knowledge about local actors, resource, interests, potential resistances, and opportunities, facilitating better work and quicker feedback within local parts of the system; (b) it can promote innovation and experimental learning, with lessons that can then be transferred to other parts of the system; and (c) it can improve local presence and risk management through local redundancy of actors and actions, strengthening the system's overall ability to deliver the envisioned changes in all parts of the system. Building *adaptive capacity*, supporting networked governance, and building leadership of people and organizations implementing the interventions are central pieces to achieve optimum polycentric governance that is transparent about the best levels of decision‐making for different problems and that has mechanisms and means to facilitate collective action and coordination.^11^

Finally, it is necessary to secure resources and accountability for scaling and sustainability. Solutions to address the rising levels of childhood obesity among US Latin*x* and Latin American populations invest a lot of their resources in designing, implementing, and evaluating the interventions, while the structures and mechanisms needed for countering intervention or policy resistance and achieving long‐term sustainability of the system's new form and function—including capacity to respond and adapt to unpredictable changes in context—oftentimes receive little consideration.^3^, ^61^ Appropriate resourcing and attention to scaling and sustainability aspects help to ensure that the system reconfiguration planned and initiated by those developing the interventions can be fully fulfilled and maintained; otherwise, the gains can be partially or totally lost over time as the system returns to its original state.

## 3 WAY FORWARD

Although awareness of and support for *systems science* in childhood obesity prevention among US Latin*x* and Latin American populations is growing, adoption of systems approaches among intervention researchers in childhood obesity has been slow.^62^, ^63^ Over 100 challenges to implementation of *systems science* in public health were identified by Trochim et al.^62^ many of which remain today.^5^ This paper has aimed to describe concrete actions to adopt *systems science* approaches applicable to the prevention of childhood obesity in US Latin*x* and Latin American populations. But wider adoption of systems‐based solutions to reduce childhood obesity in general and among US Latin*x* and Latin American populations in particular still faces many barriers,^62^, ^64^ such as (a) lack of funding resources and incentives for employing *systems science* approaches; (b) lack of an understanding of or comfort with systems measures and models, with an orientation toward non‐contextual models of intervention research, that is, randomized control trials and laboratory‐based research designs; (c) limitations in time and commitment to foster systems‐based planning and evaluation; (d) preference for predictable (hypothesized) and often short‐term outcomes we can objectively measure; (f) oversimplification of complexity in ways that undermine such contextual approaches; and (g) research and academic systems (funding timelines, academic reward systems, tenure, and promotion) that reinforce non‐system oriented research questions and designs.

At the core of moving *systems science* forward in the childhood obesity prevention field in US Latin*x* and Latin American populations is the need to build capacity in research, community, policy, and practice.^65^ New ways of working based on *systems thinking* should be a much more sustainable model of addressing childhood obesity than traditional siloed models. For instance, Pérez‐Escamilla et al.^1^ observed that interest groups playing to fears of the public concerning loss of economic opportunities during the weekly street closures can negatively affect the implementation of *Ciclovías Recreativas* throughout Latin America. People trained in systems approaches could help to embrace the perspective and goals of these interest groups in the systems map and encourage bi‐directional relationships and learning necessary for the development of shared goals and coordinated actions for the collective good, reducing the risk of intervention or policy resistance. Finally, continuous work led by and involving Latin American scholars and stakeholders will help to refine and adapt the current framework to the types of policies and interventions that are being applied and evaluated in Latin American countries.

## 4 CONCLUSION

This bold and urgent call to address childhood obesity in US Latin*x* and Latin American populations using *systems science* paves a new way toward holistic and interdisciplinary research and action. Growing evidence and interest support applications of this type of systems‐based, action‐oriented framework to reduce childhood obesity given the limited progress that has been achieved. Cross‐sectoral and cross‐disciplinary collaborations, training opportunities, a research culture open to embracing *systems science*, and funding mechanisms that support the application of the framework are needed to advance public health efforts to stem the rise in obesity in US Latin*x* and Latin American populations and promote health equity.

## CONFLICT OF INTEREST

The authors declare no conflict of interest.

## Supporting information

**Table S1.** Actions, outputs, and examples of methods and approaches for systems‐based solutions for childhood obesity prevention in U.S. Latin*x* and Latin American populations.
