# Supplementary material for: An action‐oriented framework for systems‐based solutions aimed at childhood obesity prevention in US Latinx and Latin American populations
Source: Obes Rev. 2021 Apr 7;22(Suppl 3):e13241. doi: 10.1111/obr.13241 (PMC8217154; doi:10.1111/obr.13241)
Supplement: Supplementary file 1 — Table S1. Actions, outputs, and examples of methods and approaches for systems‐based solutions for childhood obesity prevention in U.S. Latinx and Latin American populations. [file OBR-22-e13241-s001.docx]

| **Table S1.** Actions, outputs, and examples of methods and approaches for systems-based solutions for childhood obesity prevention in U.S. Latin*x* and Latin American populations. | | | |
| --- | --- | --- | --- |
| **Cog** | **Actions** | **Outputs** | **Examples of methods and approaches** |
| Foster multisectoral team | - Identify stakeholders and relationships  - Assemble a coalition  - Build trust, respect, and cohesion  - Define shared principles and aspirations | - Solid coalition of partners  - Community capacity initiated  - Leadership and direction  - Strategic roadmap  - Culture and environment that encourage collective learning, creation, and action | - Snowball engagement  - Team building best practices  - Community Coalition Action Theory  - Network analysis  - Interview key informants |
| Map the system, its context, and drivers | - Articulate the problem  - Map factors and concepts involved in the problem, their interconnections, and how they have been changing over time | -Collectively generated model of the problem space  - Shared mental model of the system  - Understanding of how the system has evolved so far and in response to what mechanisms and events | - Interview key informants  - Group model building  - Causal loop diagram |
| Envision system-wide changes | - Co-create shared vision of future  - Identify potential points of action and resistance  - Map and review evidence, lessons, and practices  - Co-design actions  - Secure resources and accountability  - Agree implementation roadmap | - Vision of future  - Theory of change  - Set of informed, collective, coordinated actions  - Implementation roadmap | - Group model building  - Structured decision making  - Scenario modelling  - Adaptive policy approaches  - Evidence-based practice approaches  - Knowledge-to-action framework  - Public participation approaches |
| Effect system-wide changes | - Implementation of coordinate actions by multisectoral team  - Active engagement to increase reach and adoption within the system | - Changes in the drivers of the problem and in the form and function of the system  - Buy-in by target population  - Adoption of new habits  - New social norms | - Evidence-based practice approaches  - Knowledge-to-action framework  - Public participation approaches  - Consolidated Framework for Implementation Research  - Diffusion of innovation  - Reach, Effectiveness, Adoption, Implementation, and Maintenance (RE-AIM) framework  - Adaptive management |
| Monitor, learn, and adapt | - Multipronged data collection  - Data analysis and interpretation  - Communicate findings  - Elicit collective lessons  - Identify areas for strengthening  - Adapt implementation roadmap | - Monitoring of the implementation  - Lessons on how the system is reacting to changes, why, and for whom  - Updated shared mental model  - Tuning and adaptation of implementation roadmap | - Network analysis  - Knowledge-to-action framework  - Public participation approaches  - Adaptive management  - Data capture  - Generative causation methods  - Statistical approaches  - Qualitative comparative analysis |
| Scale and sustain | - Define adaptation tipping points and adaptation pathways  - Secure resources and accountability  - Enable self-organization and decentralization of decision making | - System’s new form and function  - Incorporation of new habits and norms  - Process in place to avoid returning to undesired condition and control unintended consequences | - Scenario modelling  - Adaptive policy approaches  - Public participation approaches  - Networked governance |
